# Cannabis and psychosis: minimising harm while maximising therapeutic potential

**DOI:** 10.1192/bjp.2025.10389

**Published:** 2026-04

**Authors:** Rashmi Patel

**Affiliations:** Department of Psychiatry, University of Cambridge, UK

**Keywords:** Psychotic disorders/schizophrenia, substance use disorders, addictions, general adult psychiatry, epidemiology

## Abstract

Cannabis use increases the risk of psychosis, but cannabis-based medicinal products may provide additional therapeutic opportunities. Decriminalisation of cannabis has led to wider availability in certain jurisdictions, while in the UK regulated medicinal preparations are not readily accessible. A more balanced approach could reduce harms while maximising potential therapeutic benefits.

Cannabis use is associated with a two- to threefold increase in the incidence rate of schizophrenia,^1^ and with worse clinical outcomes in people with established psychotic disorders including antipsychotic treatment failure and relapse.^2^ Epidemiological studies have demonstrated that earlier use of cannabis (particularly during adolescence), higher tetrahydrocannabinol (THC) content and increased frequency of use are associated with an even greater risk of developing psychosis.^1^ In the past few decades, the concentration of THC in consumed natural cannabis has progressively increased.^3^ Meanwhile, some countries have decriminalised the recreational use of cannabis leading to an increase in the number of people diagnosed with cannabis use disorders.^4^ Given the established link between cannabis use and risk of psychotic disorders, and the increased THC concentration of cannabis being consumed in recent years, the wider availability of cannabis in certain jurisdictions raises the concern of potentially greater rates of psychosis and other non-psychotic mental disorders among individuals who are at risk and who may be able to more easily obtain high-potency cannabis.

On the other hand, cannabis may hold therapeutic potential for people with psychotic disorders. Cannabidiol (CBD), a naturally occurring cannabinoid that does not lead to intoxication when consumed, is currently being investigated as an adjunctive treatment for psychosis, although some studies have demonstrated efficacy while others have not.^5^ Herbal preparations of CBD are lawfully sold in the UK (commonly in the form of oil, capsules or edible products) without a prescription. Cannabis-based medicinal products have been developed and licensed for the treatment of other health disorders, including multiple sclerosis and treatment-resistant epilepsy, and may be obtained with a prescription in the UK. However, access to these therapies in UK National Health Service (NHS) providers has been limited, with prescriptions available only as second-line treatments for a limited range of indications from approved hospital specialists. National Institute for Health and Care Excellence (NICE) guidelines have recommended that cannabis-based medicinal products may be considered for certain indications, including intractable chemotherapy-induced nausea and vomiting, severe spasticity in adults with multiple sclerosis and severe treatment-resistant epilepsy associated with tuberous sclerosis complex, Lennox–Gastaut syndrome and Dravet syndrome. However, NICE guidelines recommend against the use of cannabis-based medicinal products for chronic pain.^6^ Cannabis-based medicinal products were legalised in the UK in November 2018 but, by 2020, only a handful of NHS prescriptions had been issued in comparison with several million people using unregulated and potentially harmful recreational cannabis.^7^

To date, there is a lack of high-quality clinical research aimed at evaluating the safety and efficacy of either herbal CBD preparations or cannabis-based medicinal products for psychotic disorders. A further therapeutic opportunity relates to the repurposing of existing cannabis-based medicinal products to provide safer alternatives to high-potency cannabis for people with comorbid cannabis use disorders, to manage withdrawal symptoms and to support safe reduction or cessation of cannabis use but, to date, little research has been conducted in this area. Even if clinical trials of cannabinoids demonstrate safety and efficacy for the treatment of psychotic disorders, or to manage cannabis use disorder and withdrawal syndromes, without approval from NICE to support the prescription of cannabis-based medicinal products for these disorders, and the necessary budget, training and infrastructure to support their prescription by psychiatrists delivering secondary mental healthcare, it is unlikely that many people with psychosis would be able to access them in the UK. At the same time, the wider availability of high-potency cannabis has the potential to harm more people who are at risk of, or already have, established psychotic disorders. A more balanced approach to shaping the clinical landscape would help to minimise harms from natural cannabis while maximising the potential therapeutic benefits of cannabis-based medicinal products.

One area that could help to inform future policy for cannabis is the recent approach to limit the nicotine content of consumed tobacco. Typical cigarettes contain up to 16 mg/g of nicotine; in contrast, very-low-nicotine-content (VLNC) cigarettes contain just 0.4 mg/g. Reducing the nicotine content of cigarettes reduces the likelihood of developing nicotine dependence, and consequently reduces exposure to the harmful effects of tobacco through reduced consumption.^8^ In the same vein, restricting the THC content of lawfully distributed cannabis in jurisdictions where recreational cannabis has been decriminalised could help to limit exposure to the harmful effects of cannabis and reduce the risk of dependence. However, to be successful, a decriminalised and regulated market for cannabis would have to outcompete a pre-existing illicit market. In New Zealand, where a recent referendum narrowly rejected a proposal to legalise the sale of cannabis, a survey of university students on perceived facilitators and barriers to switching from an illegal to a legal source of cannabis found that perceived safety of known THC content, the availability of a legal source and accessibility to a more diverse range of products were considered to be facilitators. However, limits on THC quantity and loyalty to existing dealers were considered barriers to switching from an illegal to a legal source.^9^ Such studies highlight the complexity of implementing a legal route to the distribution and selling of cannabis alongside an already established illicit market.

The greater availability of high-potency, illicitly traded cannabis poses an ongoing risk to population mental health, particularly among younger users such as adolescents. Alongside measures to minimise harms from recreational cannabis use, greater investment in clinical development and changes in guidelines, financial support, training and infrastructure – to enable wider access to regulated cannabis-based medicinal products in the UK – could help to broaden the therapeutic repertoire and provide safer options, such as cannabinoid replacement therapies, to reduce harms and potentially achieve abstinence in people with comorbid cannabis use disorders.
